# Winter Temperature Inversions and Emergency Department Visits for Asthma in Salt Lake County, Utah, 2003–2008

**DOI:** 10.1289/ehp.1104349

**Published:** 2012-07-11

**Authors:** John D. Beard, Celeste Beck, Randall Graham, Steven C. Packham, Monica Traphagan, Rebecca T. Giles, John G. Morgan

**Affiliations:** 1 Environmental Epidemiology Program, and; 2 Asthma Program, Utah Department of Health, Salt Lake City, Utah, USA; 3 National Weather Service, Salt Lake City, Utah, USA; 4 Division of Air Quality, Utah Department of Environmental Quality, Salt Lake City, Utah, USA; 5 Office of Health Care Statistics, Utah Department of Health, Salt Lake City, Utah, USA

**Keywords:** asthma, case-crossover, emergency department, interaction, inversion, winter

## Abstract

Background: Winter temperature inversions—layers of air in which temperature increases with altitude—trap air pollutants and lead to higher pollutant concentrations. Previous studies have evaluated associations between pollutants and emergency department (ED) visits for asthma, but none have considered inversions as independent risk factors for ED visits for asthma.

Objective: We aimed to assess associations between winter inversions and ED visits for asthma in Salt Lake County, Utah.

Methods: We obtained electronic records of ED visits for asthma and data on inversions, weather, and air pollutants for Salt Lake County, Utah, during the winters of 2003 through 2004 to 2007 through 2008. We identified 3,425 ED visits using a primary diagnosis of asthma. We used a time-stratified case-crossover design, and conditional logistic regression models to calculate odds ratios (ORs) and 95% confidence intervals (CIs) to estimate rate ratios of ED visits for asthma in relation to inversions during a 4-day lag period and prolonged inversions. We evaluated interactions between inversions and weather and pollutants.

Results: After adjusting for dew point and mean temperatures, the OR for ED visits for asthma associated with inversions 0–3 days before the visit compared with no inversions during the lag period was 1.14 (95% CI: 1.00, 1.30). The OR for each 1-day increase in the number of inversion days during the lag period was 1.03 (95% CI: 1.00, 1.07). Associations were only apparent when PM_10_ and maximum and mean temperatures were above median levels.

Conclusions: Our results provide evidence that winter inversions are associated with increased rates of ED visits for asthma.

Weather patterns and topographical features in Salt Lake County, Utah, are optimal for the formation of winter temperature inversions (hereafter referred to as inversions) (Gillies et al. 2010), which occur when cold air becomes trapped at the earth’s surface beneath a layer of warmer air above the surface. Bailey et al. (2011) estimated that Salt Lake City experienced daytime inversions on 57% of winter days from 1994 through 2008, although the number of inversion days varied greatly from year to year. Inversions can result in stagnant air masses and in the accumulation of air pollutants (Wallace and Kanaroglou 2009; Wallace et al. 2010), primarily particulate matter (Gillies et al. 2010; Monn et al. 1995; Silva et al. 2007; Tran and Mölders 2011) that can exceed health-based National Ambient Air Quality Standards (NAAQS) [U.S. Environmental Protection Agency (EPA) 2009b]. Salt Lake County, which covers 742 square miles and is located in a valley surrounded by mountains, is home to an estimated 1.03 million people (U.S. Census Bureau 2011) who may be involuntarily exposed to inversions and associated build-up of air pollutants.

Several studies have evaluated associations between air pollutants and adverse respiratory symptoms, including nonadmitting emergency department (ED) visits, among asthmatics (U.S. EPA 2006, 2009a).

Abdul-Wahab et al. (2005) conducted an ecologic study of < 2,500 ED visits for diseases associated with air pollution (in aggregate) that occurred during a 1-year period in Oman. They found an association between the monthly number of inversion days and the monthly mean daily number of ED visits. Wallace et al. (2010) found a cross-sectional association between inversions and airway inflammation, which was measured by sputum cell counts, among 674 persons with asthma who resided in Hamilton, Ontario, Canada, but they found no associations with individual pollutants. Previous studies have not considered inversions or days on which inversions occurred as independent variables contributing to rates of ED visits for asthma.

We conducted a study of the effect of winter inversions on ED visits for asthma to provide data upon which doctors, patients, and public health officials can base inversion-specific asthma management plans and asthma-trigger prevention messages. Because inversions occur primarily during the winter months in Salt Lake County (Gillies et al. 2010), we used data from the winter months, December through February, from 2003–2004 to 2007–2008. Analytically, we aimed to describe inversions in terms of weather and pollutant concentrations, to estimate associations between ED visits for asthma and the occurrence of inversions and the occurrence of prolonged inversions, and to determine whether associations between inversions and ED visits for asthma changed when we adjusted for concentrations of individual pollutants that often occur during inversions.

## Methods

*Inversion criteria*. The National Weather Service (NWS) office in Salt Lake City is one of approximately 90 sites in North America that routinely launch weather balloons (frequently referred to as upper-air soundings) as part of the NWS Upper-air Observations Program (NWS 2009). Weather balloons are launched twice each day, shortly after 0400 hours and after 1600 hours Mountain Standard Time; they gather temperature, humidity, and wind data as they ascend through the troposphere. Analysis of data gathered by weather balloons is one of the primary methods used by meteorologists at the NWS to identify the presence, and the strength, of inversions. We examined all data gathered from upper-air soundings released by the NWS office in Salt Lake City to identify inversion events in Salt Lake County during the study period (University of Wyoming 2012). The American Meteorological Society defines inversions as layers in which “temperature increases with altitude” (Glickman 2000) instead of following the normal pattern of decreasing air temperature with increasing altitude. For this study, we required that the bottom of the inversion (i.e., the height of the air layer where the temperature begins to increase with increasing altitude) had to be below the crest level of the surrounding mountains (within 5,000–7,000 ft of the valley floor). This requirement ensured inversions were low enough to potentially trap pollutants near ground level. The bottom of the inversion could be at ground level or above ground level as long as the bottom was below the crest of the surrounding mountains. Additionally, we only considered inversions that were present at upper-air soundings at both 0400 hours and 1600 hours on a given day to exclude short-term inversions in the morning due to intense radiational cooling overnight (Gillies et al. 2010). In our study, we did not account for inversion depth (the altitude difference from the bottom to the top of the inversion) or strength (the temperature difference from the bottom to the top of the inversion).

*Data*. We obtained an electronic, limited data set of daily ED visits for asthma at all 10 Salt Lake County hospitals from the Office of Health Care Statistics, Utah Department of Health (2012a). One hospital was open from the beginning of the study period until it closed during the summer of 2007. Another hospital opened during the summer of 2007 to replace the one that closed and remained open through the end of the study period. The other 8 hospitals were open during the entire study period. The ED records, which represented complete coverage for Salt Lake County, included ED visit identification numbers assigned by the Office of Health Care Statistics, Utah Department of Health (2012b), age, sex, race, Hispanic ethnicity, *International Classification of Diseases, 9th Revision* (ICD-9) (World Health Organization 1977) diagnosis codes, date of admission, and residential ZIP code. No identifying information, such as names or birth dates, was included in the ED records. We included all residents living within Salt Lake County ZIP codes who had visited the ED for asthma, identified by an ICD-9 primary diagnosis code beginning with “493,” during the study period. Data on race and Hispanic ethnicity were missing for 32% and 53%, respectively, of records; thus, we dropped those covariates from further analyses.

We downloaded GSOD (global summary of day) measurements of daily maximum, mean (of maximum and minimum), and dew point temperatures (degrees Fahrenheit) for the Salt Lake City International Airport (one site) from the Climate Database server on the Utah Climate Center website (Utah State University 2012) and converted them to degrees Celsius. Measurements were missing for < 1% (1 of 452) of study days, so we imputed them by averaging measurements from days before and after missing days. We derived relative humidity (percent) from mean and dew point temperatures using the Magnus formula (see Lawrence 2005, Equation 8), solved for relative humidity, and constants suggested by Alduchov and Eskridge (1996): *a* = 17.625, *b* = 243.04. We obtained upper-air soundings data from the University of Wyoming (2012).

We obtained data on six air pollutants from the U.S. EPA AirData (2012) website: carbon monoxide (CO, four monitors), nitrogen dioxide (NO_2_, two monitors), ozone (O_3_, one monitor), particulate matter (PM) with an aerodynamic diameter ≤ 2.5 µm (PM_2.5_, seven monitors) and ≤ 10 µm (PM_10_, four monitors), and sulfur dioxide (SO_2_, three monitors). Data on O_3_ were available only for 1 January 2006 through 29 February 2008 (240 of 452 study days; 53%). For CO, NO_2_, PM_2.5_, PM_10_, and SO_2_, we assigned exposures to residents based on concentrations measured by the monitor nearest to the centroid of their residential ZIP code. Monitors were located where pollution levels were expected to be the highest (CO, two monitors; NO_2_, two monitors; O_3_, one monitor; PM_2.5_, two monitors; PM_10_, three monitors; SO_2_, two monitors) and to establish ambient levels of pollutants (CO, two monitors; PM_2.5_, five monitors; PM_10_, one monitor). Monitors were also placed next to specific industry sources to determine pollutant concentrations (SO_2_, one monitor) (Utah Department of Environmental Quality 2006, 2011). We used daily 1-hr maximum concentrations for CO, NO_2_, O_3_, and SO_2_, and 24-hr mean concentrations for PM_2.5_ and PM_10_. Concentrations for some days were missing for PM_2.5_ (5 of 452 days; 1%), PM_10_ (3 of 452; 1%), and O_3_ (7 of 240; 3%). Thus, we imputed them by averaging concentrations measured on the day before and the day after each missing day.

*Statistical analyses*. We employed a time-stratified case-crossover design (Levy et al. 2001), which uses cases as their own controls (Maclure 1991). Each resident who visited an ED for asthma during the study period was assigned a case day based on the day of the visit, and control days on the same day of the week for all other weeks during the month in which the ED visit occurred, regardless of whether the control days occurred before or after the ED visit (Levy et al. 2001). If a single person completed a second ED visit for asthma within 2 days, we counted the first visit only and assigned control days according to the day of the first visit. If a single person completed a second ED visit for asthma on a control day, that is, on the same day of the week during the same month (1% of all cases in our study), we counted the second visit as both a case day and a control day. In total, we included 3,425 case days and 11,473 control days in our analysis. We also conducted a sensitivity analysis that excluded residents who completed a second ED visit for asthma on one of their control days (i.e., the 1% of cases referred to above). Before we obtained the limited data set we used for this analysis, residents who completed a second ED visit for asthma within 2 days or on one of their control days were identified by the Utah Department of Health.

We used conditional logistic regression models to estimate odds ratios (ORs) and 95% confidence intervals (CIs) for the association between inversions and ED visits for asthma. We used an *a priori* lag structure of 0–3 days (the day of the ED visit for asthma and the 3 days before the ED visit for case days, and each of the corresponding control days and the 3 days before for control days). We used constrained quadratic polynomial distributed lag models to estimate lag-specific and overall effects of inversions relative to noninversions on ED visits for asthma (Schwartz 2000). We also fit unconstrained distributed lag and 4-day moving average models to evaluate the robustness of our results to our choice of model. All model covariates were represented by 4-day moving averages unless otherwise specified. Models with weather and pollutant variables coded using quadratic, cubic, or spline terms did not significantly improve model fit based on likelihood ratio tests (α = 0.05). Thus, all covariates were modeled as simple continuous variables. We adjusted all models for dew point and mean temperatures. In addition, we ran separate models adjusted for each of the six air pollutants one at a time to determine whether adjusting for pollutants altered associations between inversions and ED visits for asthma.

We modeled dose–response relations according to the length of inversions in three ways. First, we categorized the number of inversion days during lag 0–3 as zero (reference), 1, 2, 3, or 4 inversion days during the lag period. Second, we categorized inversions according to the day of inversion [the number of consecutive inversion days that had occurred on or before the case (ED visit) or corresponding control days] as not an inversion day (reference), 1st–3rd, 4th–6th, 7th–12th, or > 12th day of an inversion. Third, we categorized the inversion length based on the number of consecutive inversion days up to and including case or control days as not an inversion (reference), 1–3, 4–6, 7–8, or > 8 days. We tested linear trends by modeling ordinal versions of the three dose–response variables coded as the number of inversion days for the first variable and by using category medians for the day of inversion and inversion length variables.

To evaluate interactions between inversions and age, sex, weather, and pollutants, we modeled dichotomized variables and product terms as any inversion days during lag 0–3 [no (reference), yes], age [≤ 34 (reference), > 34 years of age], sex [male (reference), female], weather or pollutants [≤ median value (reference), > median]. We tested the significance of multiplicative interactions using likelihood ratio tests comparing models with and without product terms with α = 0.1.

We performed the following sensitivity analyses: excluded individuals who resided in Salt Lake County, but visited an ED for asthma outside of Salt Lake County (3% of ED visits); excluded individuals with ED visits associated with acute respiratory infections (ICD-9 codes 460.0–466.0; 16% of ED visits) in addition to asthma; used average air pollutant concentrations across all available monitors in Salt Lake County instead of using concentrations from monitors closest to residences (for pollutants other than O_3_, which was measured at only one site); adjusted for relative humidity instead of dew point temperature; and adjusted for maximum temperature rather than daily mean temperature. As noted previously, we also repeated models after adjusting for individual air pollutants to determine whether adjusting for these potential causal intermediates altered associations between inversions and ED visits for asthma. We conducted all analyses with SAS software (version 9.2; SAS Institute Inc., Cary, NC) using PROC LOGISTIC to fit conditional logistic regression models. We obtained Institutional Review Board approval from the Utah Department of Health; our study was exempted from informed consent requirements because our study was a secondary data analysis of preexisting data.

## Results

A mean (± SD) of 8.11 ± 3.40 ED visits for asthma occurred each day during our study period, and more ED visits for asthma occurred on inversion days than on noninversion days (8.41 ± 3.52 versus 7.94 ± 3.32 visits/day; *p* < 0.0001) (Table 1). The mean number of ED visits also increased, although not monotonically, by day of inversion (*p* < 0.0001) and was higher after 12 inversion days (11.42 ± 2.76 visits/day) compared with the first 3 days of an inversion (7.98 ± 3.45 visits/day) (Table 1). Females accounted for 54% of ED visits, and the percentage of visits by residents 0–4, 5–17, 18–34, 35–54, and > 54 years of age was 25%, 20%, 24%, 21%, and 10%, respectively. Of the 452 study days, inversions occurred on 158 (35%) days during the study period, which yielded 5,217 person-days during inversions and 9,681 person-days during noninversions among residents included in the analysis because of an ED visit for asthma (Table 1). O_3_ concentrations were significantly lower on inversion days compared with noninversion days, whereas concentrations of the other pollutants evaluated were significantly higher (Table 1). Concentrations of NO_2_, PM_2.5_, and PM_10_ increased as day of inversion increased, but there was no strong evidence of this for CO or SO_2_ (Table 1). O_3_ concentrations also increased as day of inversion increased, but this increase was toward the levels observed during noninversion days instead of away from them (Table 1). Inversion days were also colder and more humid than were noninversion days (Table 1). Descriptive statistics for weather variables and pollutant concentrations during the entire study period are shown in Supplemental Material, Table S1 (http://dx.doi.org/10.1289/ehp.1104349).

**Table 1:** Daily ED visits for asthma, ambient air quality, and weather by inversions for Salt Lake County, Utah, December through February, 2003–2004 to 2007–2008.

Variable (units)c	Total	Inversiona			Day of inversionb						
		No	Yes	1st–3rd	4th–6th	7th–12th	> 12th
Days [no. (%)]	452	294 (65)	158 (35)	105 (23)	36 (8)	11 (2)	6 (1)
ED visits for asthma [no. (%)]	3,425	2,191 (64)	1,234 (36)	774 (23)	319 (9)	75 (2)	66 (2)
Person-days [no. (%)]	14,898	9,681 (65)	5,217 (35)	3,385 (23)	1,221 (8)	380 (3)	231 (2)
Daily no. ED visits for asthma (mean ± SD)	8.11 ± 3.40	7.94 ± 3.32	8.41 ± 3.52*	7.98 ± 3.45	9.29 ± 3.26	7.58 ± 3.78	11.42 ± 2.76*
CO (ppm, mean ± SD)	1.35 ± 0.70	1.12 ± 0.58	1.76 ± 0.70*	1.79 ± 0.68	1.83 ± 0.81	1.58 ± 0.51	1.24 ± 0.33*
NO2 (ppb, mean ± SD)	49.08 ± 12.88	44.91 ± 10.21	56.83 ± 13.73*	54.91 ± 12.95	58.37 ± 15.47	64.40 ± 13.08	64.35 ± 5.83*
O3 (ppb, mean ± SD)d	23.09 ± 10.14	26.24 ± 8.33	14.17 ± 9.47*	12.59 ± 8.22	18.18 ± 12.27	20.20 ± 4.86*	NAe
PM2.5 (µg/m3, mean ± SD)	21.04 ± 18.21	12.23 ± 10.22	37.39 ± 18.48*	32.97 ± 15.42	40.86 ± 21.42	51.39 ± 17.03	60.80 ± 10.18*
PM10 (µg/m3, mean ± SD)	36.02 ± 24.31	24.41 ± 16.20	57.59 ± 22.01*	54.20 ± 19.65	59.79 ± 26.67	70.53 ± 19.09	74.26 ± 13.40*
SO2 (ppb, mean ± SD)	5.72 ± 5.35	4.68 ± 4.83	7.66 ± 5.71*	7.36 ± 5.76	8.35 ± 5.96	7.98 ± 5.04	7.74 ± 4.24*
Dew point temperature (°C, mean ± SD)	–5.33 ± 4.12	–4.81 ± 4.09	–6.28 ± 4.01*	–6.16 ± 4.32	–5.46 ± 3.29	–8.15 ± 2.83	–9.33 ± 1.17*
Maximum temperature (°C, mean ± SD)	4.28 ± 5.26	5.28 ± 5.22	2.42 ± 4.81*	3.07 ± 4.71	2.97 ± 4.37	–1.53 ± 4.11	–3.50 ± 0.62*
Mean temperature (°C, mean ± SD)	–0.93 ± 4.65	0.08 ± 4.63	–2.80 ± 4.07*	–2.44 ± 4.18	–2.15 ± 3.51	–5.60 ± 3.42	–6.88 ± 1.12*
Relative humidity (%, mean ± SD)f	73.05 ± 10.81	70.69 ± 11.01	77.43 ± 8.88*	76.04 ± 8.62	78.62 ± 9.17	82.84 ± 9.00	82.67 ± 2.31*
aDifferences between values on inversion days versus noninversion days tested via Wilcoxon rank sum tests. bDifferences between values across the four levels of day of inversion tested via Kruskal–Wallis tests. cThe daily 1-hr maximum was used for CO, NO2, O3, SO2, and maximum temperature, and the 24-hr mean was used for PM2.5, PM10, dew point temperature, and mean temperature. dData on O3 were available only for 1 January 2006 through 29 February 2008. eNo inversion occurring after 1 January 2006 (when data on O3 were available) lasted longer than 8 days. fRelative humidity was derived from dew point temperature and mean temperature using the Magnus formula (see Lawrence 2005, Equation 8) but solved for relative humidity, with the constants suggested by Alduchov and Eskridge (1996). *p < 0.0001.														

The OR for ED visits for asthma in association with inversions versus no inversions during lag 0–3, based on constrained quadratic polynomial distributed lag models adjusted for dew point and mean temperatures (Model 1), was 1.14 (95% CI: 1.00, 1.30) (Table 2). The strongest association with inversions on a specific lag day was for lag 0 (OR = 1.05; 95% CI: 0.95, 1.16) (Table 2). Results from unconstrained distributed lag models and 4-day moving average models were similar [see Supplemental Material, Table S2 (http://dx.doi.org/10.1289/ehp.1104349)].

**Table 2:** ORs and 95% CIs for ED visits for asthma and inversions for Salt Lake County, Utah, December through February, 2003–2004 to 2007–2008.

Variable	Model 1 OR^a,b^ (95% CI)	Model 2 OR^a,c^ (95% CI)
Constrained quadratic polynomial distributed lag models		
Inversion
Lag 0
No	Reference	Reference
Yes	1.05 (0.95, 1.16)	1.05 (0.95, 1.16)
Lag 1
No	Reference	Reference
Yes	1.02 (0.95, 1.09)	1.02 (0.94, 1.10)
Lag 2
No	Reference	Reference
Yes	1.02 (0.95, 1.09)	1.01 (0.94, 1.09)
Lag 3
No	Reference	Reference
Yes	1.04 (0.95, 1.15)	1.03 (0.93, 1.14)
Overall
No	Reference	Reference
Yes	1.14 (1.00, 1.30)	1.12 (0.93, 1.33)
No. of inversion days during lag 0–3		
0	Reference	Reference
1	1.06 (0.94, 1.20)	1.06 (0.94, 1.20)
2	0.99 (0.87, 1.12)	0.98 (0.86, 1.12)
3	1.10 (0.96, 1.25)	1.08 (0.93, 1.27)
4	1.17 (1.01, 1.35)	1.14 (0.95, 1.39)
Trendd	1.03 (1.00, 1.07)	1.03 (0.98, 1.07)
Day of inversion		
No inversion	Reference	Reference
1st–3rd	1.05 (0.95, 1.16)	1.04 (0.94, 1.16)
4th–6th	1.19 (1.03, 1.37)	1.17 (0.99, 1.38)
7th–12th	0.88 (0.67, 1.15)	0.86 (0.65, 1.15)
> 12th	1.37 (1.01, 1.85)	1.33 (0.97, 1.84)
Trende	1.02 (1.00, 1.04)	1.02 (1.00, 1.04)
Inversion length (days)		
No inversion	Reference	Reference
1–3	1.16 (1.02, 1.33)	1.15 (1.01, 1.31)
4–6	0.99 (0.87, 1.13)	0.96 (0.84, 1.11)
7–8	1.16 (0.97, 1.39)	1.11 (0.92, 1.34)
> 8	0.95 (0.74, 1.22)	0.89 (0.67, 1.16)
Trendf	1.00 (0.99, 1.01)	1.00 (0.98, 1.01)
aThe 24-hr mean was used for PM2.5, dew point temperature, and mean temperature. bAdjusted for dew point temperature and mean temperature. cAdjusted for dew point temperature, mean temperature, and PM2.5. dOR for ordinal variable coded as 0, 1, 2, 3, or 4. eOR for ordinal variable coded using the following median category scores: 0, 2, 5, 8, 15. fOR for ordinal variable coded using the following median category scores: 0, 2, 5, 8, 18.		

When estimated according to the number of inversion days during lag 0–3, adjusted ORs were strongest for 3 and 4 inversion days [OR = 1.17 (95% CI: 1.01, 1.35) for 4 inversion days versus no inversion days during lag 0–3; adjusted OR for each additional inversion day versus no inversion day was 1.03 (95% CI: 1.00, 1.07)] (Table 2). The odds of ED visits for asthma were significantly elevated on the 4th–6th and > 12th days of inversions, but not on the 7th–12th days, compared with noninversion days, with a significant trend (OR = 1.02; 95% CI: 1.00, 1.04) (Table 2). ED visits were positively associated with inversions that had lasted for 1–3 or 7–8 days, but not with inversions that had lasted 4–6 days or > 8 days (Table 2).

Additional adjustment for PM_2.5_, the air pollutant of greatest *a priori* interest (Gillies et al. 2010), had little influence on associations between ED visits for asthma and inversion days (Table 2); adjustment for CO, NO_2_, PM_10_, and SO_2_ also showed little or no influence (data not shown). Adjusting for O_3_ also had little influence on associations when compared with ORs restricted to the time period when O_3_ data were available [see Supplemental Material, Table S3 (http://dx.doi.org/10.1289/ehp.1104349)].

Multiplicative interactions between inversions and age, sex, CO, NO_2_, O_3_, PM_2.5_, SO_2_, dew point temperature, and relative humidity were not significant at α = 0.1 (data not shown), but interactions between inversions and PM_10_, maximum temperature, and mean temperature were significant (Table 3). Specifically, positive associations between ED visits for asthma and inversions were present when PM_10_ exceeded 32.50 μg/m^3^, maximum temperature exceeded 4.03°C, or mean temperature exceeded –0.75°C, whereas inversions were not associated with ED visits when PM_10_ was ≤ 32.50, maximum temperature was ≤ 4.03, or mean temperature was ≤ –0.75 (Table 3). A similar pattern, although not statistically significant, was observed for PM_2.5_ (Table 3).

**Table 3:** Interactions from 4-day moving average models for Salt Lake County, Utah, December through February, 2003–2004 to 2007–2008.

Variables (units)^a^	Stratified adjusted OR (95% CI)^b^	p-Value for homogeneity^c^
PM2.5 (µg/m3)	Inversion days during lag 0–3
≤ 16.68	No	Reference
Yes	1.00 (0.89, 1.13)
> 16.68	No	Reference
Yes	1.11 (0.92, 1.33)	0.34
PM10 (µg/m3)	Inversion days during lag 0–3
≤ 32.50	No	Reference
Yes	0.99 (0.87, 1.11)
> 32.50	No	Reference
Yes	1.21 (1.01, 1.44)	0.04
Maximum temperature (°C)	Inversion days during lag 0–3
≤ 4.03	No	Reference
Yes	0.98 (0.86, 1.12)
> 4.03	No	Reference
Yes	1.14 (1.02, 1.27)	0.08
Mean temperature (°C)	Inversion days during lag 0–3
≤ –0.75	No	Reference
Yes	0.95 (0.83, 1.09)
> –0.75	No	Reference
	Yes	1.15 (1.03, 1.28)	0.03
aThe daily 1-hr maximum was used for maximum temperature, and the 24-hr mean was used for PM2.5, PM10, dew point temperature, and mean temperature. bAdjusted for dew point temperature and mean temperature. cInteractions tested via likelihood ratio tests that compared the model including the interaction product term with the model excluding it.						

Excluding those cases who returned to the ED for asthma on one of their control days (*n* = 38, 1%) or who resided in Salt Lake County but visited an ED for asthma outside of Salt Lake County (*n* = 92, 3%) gave nearly identical results (data not shown). Excluding 538 (16%) individuals who had acute respiratory infections at the time of their ED visits for asthma increased the ORs slightly (e.g., adjusted OR = 1.21; 95% CI: 1.04,1.40 for any inversion days during lag 0–3 compared with OR = 1.14; 95% CI: 1.00, 1.30). Using average pollutant concentrations across all available monitors in Salt Lake County instead of using concentrations from monitors closest to residences, adjusting for relative humidity instead of dew point temperature, and adjusting for maximum temperature in models instead of mean temperature gave nearly identical results (data not shown).

## Discussion

Pollutant concentrations and the adjusted odds of ED visits for asthma were higher during inversions than noninversions, with stronger associations as the number of inversion days increased during the lag period. Results suggested that associations were stronger for longer inversions, although ORs did not increase monotonically. Although air pollutant concentrations changed during inversions (decreasing for O_3_ and increasing for all other pollutants on inversion days versus noninversion days), there was little change in ORs when individual air pollutant concentrations were included in models. Associations between inversion days and ED visits for asthma appeared to be limited to days with PM_10_, maximum temperature, and mean temperature above median levels based on stratum-specific ORs.

Other studies also have found increased NO_2_ (Wallace and Kanaroglou 2009), PM_2.5_ (Gillies et al. 2010; Silva et al. 2007; Tran and Mölders 2011; Wallace and Kanaroglou 2009), and PM_10_ (Monn et al. 1995) during inversions compared with noninversions. A study in Hamilton, Ontario, observed increases in NO_2_ and PM_2.5_ of 49% and 54%, respectively, during nighttime inversions (Wallace and Kanaroglou 2009). During daytime inversions, however, NO_2_ increased by only 11%, and PM_2.5_ decreased by 14%. Silva et al. (2007) studied PM_2.5_ in Cache Valley, Utah, during January–February of 2004, a period when the 24-hr NAAQS was exceeded 17 times, and found that the highest PM_2.5_ concentrations occurred during inversions. Increased PM_2.5_ concentrations during inversions were also noted in Fairbanks, Alaska, based on 10 years of data (Tran and Mölders 2011). Monn et al. (1995) measured PM_10_ during a 1-year period at 12 sites across Switzerland and noted that PM_10_ concentrations were highest during inversions.

Increased concentrations of air pollutants during inversions could be at least partly responsible for associations between inversions and ED visits for asthma observed in our study. The results of our interaction analysis support this idea because associations between inversion days and ED visits for asthma were apparent only when PM_10_ concentrations exceeded median levels and a similar pattern, although not statistically significant, was seen when we stratified by PM_2.5_. Adjusting for the six pollutants one at a time, however, did not remove the observed association. This does not preclude the possibility inversions were a marker of high concentrations of multiple air pollutants that may have been the underlying cause of the association. Furthermore, pollutant exposures were estimated using data from U.S. EPA monitors, which may have resulted in misclassification of individual exposures.

A 1-year ecologic study conducted during 2003 evaluated relationships between the occurrence of inversions and air pollution-associated diseases (in aggregate) in Oman (Abdul-Wahab et al. 2005). Monthly number of inversion days was associated with monthly mean daily number of ED visits for asthma, chronic obstructive lung diseases, heart failure, conduction and cardiac dysrhythmias, and ischemic heart disease as a combined outcome. Associations were stronger as the depth (the difference in altitudes between the top and bottom layers) and strength (the difference in temperatures between the top and bottom layers) of the inversion increased. Results were not adjusted for covariates, such as age, sex, temperature, humidity, or pollutants, and < 2,500 ED visits occurred during the study period. Wallace et al. (2010) conducted a cross-sectional study of 674 asthmatics who attended a respiratory health clinic during inversion days and noninversion days between January 2004 and December 2006 in Hamilton, Ontario. Spontaneous or induced sputum was collected from each patient at each clinic visit. Patients were classified as “exacerbated” (*n* = 189) if they were experiencing worsening asthma symptoms at the time of the clinic visit or “stable” (*n* = 485) if their asthma symptoms were under control. After adjusting for age, smoking status, corticosteroid medication, surface temperature and relative humidity, a statistically significant increase in sputum cell counts, which is indicative of airway inflammation, was associated with the occurrence of inversions, but not with individual pollutant concentrations. Specifically, neutrophil and macrophage counts were 12.6% higher in the stable group and 2.5% higher in the exacerbated group on inversion days compared with noninversion days. Oxidative stress would be a plausible biological mechanism in the scenario where inversions act as indicators of high multipollutant atmospheres (Bowler and Crapo 2002). Further research, however, is needed to determine if characteristics associated with inversions, such as increased pollutant concentrations, are the underlying cause or causes of the observed association.

Results from constrained quadratic polynomial distributed lag models suggested the association between inversions and ED visits for asthma was distributed over the entire 0–3 day lag, but strongest at lag 0. None of the lag-specific estimates were very precise, so these results should be interpreted cautiously. Dose–response relationships between inversions and ED visits for asthma were suggested when we estimated associations with the number of inversion days during the 0–3 day lag, the day of inversion, and the length of inversions, though clear monotonic relationships were not apparent.

The occurrence of multiple, prolonged winter inversions is a unique characteristic of Salt Lake County that made it possible to estimate effects of inversions on rates of ED visits for asthma. The ED billing data used in this study are likely to reflect valid diagnoses of asthma (Reeves et al. 2006; Silverman et al. 2003). Silverman et al. (2003), after conducting a 2-week review of ED patient charts and billing data in 11 New York City hospitals, reported 75% of asthma diagnoses listed in patient charts were also coded as asthma in the billing data. Reeves et al. (2006) reported similarly high concordance between asthma diagnoses listed in pediatric ED patient charts and billing data. Because we implemented a case-crossover design, each case served as his or her own control and confounding by between-person differences and covariates that do not change within individuals during the study period (sex, race) was eliminated (Carracedo-Martínez et al. 2010).

An important limitation of our study was our inability to measure individual-level exposures to inversions or pollutants. Asthmatics may have self-medicated more often or remained mostly indoors during inversions. Despite the high concordance of asthma diagnoses between billing data and ED patient charts (Reeves et al. 2006; Silverman et al. 2003), ED visits for asthma may have been misclassified, particularly on inversion days if clinicians knew of the association between air pollution and asthma and of the association between inversions and air pollution. Thus, effect measure estimates could have been biased toward or away from the null. Lower power and efficiency have been noted as limitations of case-crossover designs compared with time-series designs (Bateson and Schwartz 1999; Künzli and Schindler 2005).

## Conclusions

Our study found an association between inversions, including prolonged ones, and ED visits for asthma. This result needs to be confirmed in other populations. Further research is needed to more fully investigate the extent to which inversions act as indicators of increased air pollutant concentrations that may lead to ED visits for asthma or other health effects. Finally, the potential value of incorporating inversion forecasts and/or warnings into existing air quality alert programs should be studied in the future.

## Supplemental Material

(180 KB) PDF
