# Supplementary material for: Winter Temperature Inversions and Emergency Department Visits for Asthma in Salt Lake County, Utah, 2003–2008
Source: Environ Health Perspect. 2012 Jul 11;120(10):1385–90. doi: 10.1289/ehp.1104349 (PMC3491922; doi:10.1289/ehp.1104349)
Supplement: (180 KB) PDF [file ehp.1104349.s001.pdf]

Supplemental Material

Winter Temperature Inversions and Emergency Department Visits for Asthma in Salt Lake County, Utah, 2003-2008

John D. Beard[1], Celeste Beck[2], Randall Graham[3], Steven C. Packham[4], Monica Traphagan[3], Rebecca T. Giles[2], John G. Morgan[5]

[1]Environmental Epidemiology Program, Utah Department of Health, Salt Lake City, Utah, USA; [2]Asthma Program, Utah Department of Health, Salt Lake City, Utah, USA; [3]National Weather Service, Salt Lake City, Utah, USA;[4]Division of Air Quality, Utah Department of Environmental Quality, Salt Lake City, Utah, USA; [5]Office of Health Care Statistics, Utah Department of Health, Salt Lake City, Utah, USA

Address correspondence to J. Beard, Department of Epidemiology, Gillings School of Global Public Health, University of North Carolina, CB # 7435, 2101 McGavran-Greenberg, Chapel Hill, NC 2799-7435 USA. Telephone: (919) 797-1987. Fax: (919) 966-2089. E-mail: jdbeard@email.unc.edu

Table of Contents

Section Page Number

Supplemental Material, Table S1…………………………………………………………………..S3

Supplemental Material, Table S2…………………………………………………………………..S4

Supplemental Material, Table S3…………………………………………………………………..S5

References…………………………………………………………………………………………….S7

Supplemental Material, Table S1. Distributions of daily ambient air quality and weather for Salt Lake County, Utah, December-February, 2003-2004 to 2007-2008.

		Percentiles						
Variable[a]	Mean ± SD	Min	5th	25th	50th	75th	95th	Max
CO (ppm)	1.35 ± 0.70	0.30	0.40	0.90	1.20	1.70	2.70	5.20
NO_2 (ppb)	49.08 ± 12.88	2.00	32.00	41.00	47.00	55.00	75.00	100.00
O_3[b] (ppb)	23.09 ± 10.14	2.00	6.00	15.00	25.00	31.00	38.00	43.00
$PM_{2.5}$ (µg/m^3)	21.04 ± 18.21	0.80	2.60	7.40	14.25	31.10	60.50	94.20
PM_{10} (µg/m^3)	36.02 ± 24.31	2.00	7.00	16.00	30.00	52.00	82.00	122.00
SO_2 (ppb)	5.72 ± 5.35	0.00	1.00	2.00	4.00	8.00	16.00	40.00
Dew point temperature (°C)	-5.33 ± 4.12	-17.56	-12.72	-8.28	-5.00	-1.89	0.56	3.61
Maximum temperature (°C)	4.28 ± 5.26	-7.78	-4.00	0.00	3.89	8.28	12.78	19.00
Mean temperature (°C)	-0.93 ± 4.65	-12.89	-8.56	-4.28	-0.72	2.39	6.56	14.72
Relative humidity[c] (%)	73.05 ± 10.81	20.26	54.74	66.68	73.28	80.66	89.35	96.41

Min = minimum; Max = maximum.
a The daily 1-hour maximum was used for CO, NO_2, O_3, SO_2, and maximum temperature and the 24-hour average was used for $PM_{2.5}$, PM_{10}, dew point temperature, and mean temperature. b Data on O_3 were available only for January 1, 2006, and later. c Relative humidity was derived from dew point temperature and mean temperature using Magnus' formula (equation (8) in Lawrence 2005), but solved for relative humidity, with the constants suggested by Alduchov and Eskridge (1996): a = 17.625, b = 243.04.

Supplemental Material, Table S2. ORs and 95% CIs from unconstrained distributed lag and moving average models for Salt Lake County, Utah, December-February, 2003-2004 to 2007-2008.

Variable	Model 1 OR[a, b] (95% CI)	Model 2 OR[a, c] (95% CI)	Model 1 OR[a, b, d] (95% CI)	Model 3 OR[a, e, f] (95% CI)
Inversions				
Unconstrained distributed lag models				
Lag 0				
No	Reference	Reference	Reference	Reference
Yes	1.09 (0.98, 1.21)	1.08 (0.97, 1.21)	1.14 (0.97, 1.33)	1.21 (1.03, 1.43)
Lag 1				
No	Reference	Reference	Reference	Reference
Yes	0.95 (0.84, 1.07)	0.95 (0.84, 1.07)	0.96 (0.80, 1.15)	1.00 (0.83, 1.20)
Lag 2				
No	Reference	Reference	Reference	Reference
Yes	1.09 (0.97, 1.23)	1.09 (0.96, 1.23)	1.12 (0.94, 1.34)	1.15 (0.96, 1.38)
Lag 3				
No	Reference	Reference	Reference	Reference
Yes	1.01 (0.91, 1.12)	1.00 (0.90, 1.12)	1.00 (0.86, 1.17)	1.02 (0.87, 1.18)
Overall				
No	Reference	Reference	Reference	Reference
Yes	1.14 (1.00, 1.30)	1.12 (0.93, 1.33)	1.23 (1.01, 1.49)	1.42 (1.12, 1.80)
Moving average: lags 0-3	1.13 (0.99, 1.29)	1.11 (0.93, 1.32)	1.22 (1.00, 1.47)	1.37 (1.09, 1.73)

a The daily 1-hour maximum was used for O_3 and the 24-hour average was used for $PM_{2.5}$, dew point temperature, and mean temperature. b Adjusted for dew point temperature and mean temperature. c Adjusted for dew point temperature, mean temperature, and $PM_{2.5}$. d Restricted to January 1, 2006, and later. e Adjusted for dew point temperature, mean temperature, and O_3. f Data on O_3 were available only for January 1, 2006, and later.

Supplemental Material, Table S3. ORs and 95% CIs for ED visits for asthma and inversions for Salt Lake County, Utah, January-February 2006 and December-February, 2006-2007 to 2007-2008.

Variable	Model 1 OR[a, b] (95% CI)	Model 2 OR[a, c] (95% CI)
Constrained quadratic polynomial distributed lag models		
Inversion		
Lag 0		
No	Reference	Reference
Yes	1.09 (0.95, 1.26)	1.17 (1.00, 1.36)
Lag 1		
No	Reference	Reference
Yes	1.05 (0.95, 1.15)	1.09 (0.98, 1.21)
Lag 2		
No	Reference	Reference
Yes	1.03 (0.93, 1.13)	1.05 (0.95, 1.16)
Lag 3		
No	Reference	Reference
Yes	1.04 (0.91, 1.19)	1.06 (0.92, 1.21)
Overall		
No	Reference	Reference
Yes	1.23 (1.01, 1.49)	1.42 (1.12, 1.80)
No. of inversion days during lag 0-3		
0	Reference	Reference
1	1.11 (0.94, 1.31)	1.14 (0.97, 1.35)
2	0.91 (0.77, 1.08)	0.97 (0.80, 1.18)
3	1.13 (0.92, 1.40)	1.22 (0.97, 1.54)
4	1.38 (1.11, 1.71)	1.50 (1.17, 1.92)
Trend[d]	1.05 (1.00, 1.10)	1.08 (1.02, 1.15)
Day of inversion		
Not an inversion	Reference	Reference
1st-3rd	1.05 (0.91, 1.23)	1.13 (0.96, 1.34)
4th-6th	1.45 (1.16, 1.81)	1.59 (1.25, 2.02)
7th-12th	0.99 (0.62, 1.57)	1.04 (0.66, 1.66)
> 12th	NA[e]	NA[e]
Trend[f]	1.04 (1.01, 1.08)	1.06 (1.02, 1.10)

Supplemental Material, Table S3. cont.

Variable	Model 1 OR[a, b] (95% CI)	Model 2 OR[a, c] (95% CI)
Inversion length (days)		
Not an inversion	Reference	Reference
1-3	1.08 (0.91, 1.29)	1.16 (0.95, 1.41)
4-6	1.10 (0.89, 1.36)	1.16 (0.93, 1.45)
7-8	1.26 (1.02, 1.56)	1.34 (1.07, 1.67)
> 8	NA[e]	NA[e]
Trend[g]	1.03 (1.00, 1.05)	1.03 (1.01, 1.06)

a The daily 1-hour maximum was used for O_3 and the 24-hour average was used for dew point temperature, and mean temperature. b Adjusted for dew point temperature and mean temperature. c Adjusted for dew point temperature, mean temperature, and O_3.d OR for ordinal variable coded as 0, 1, 2, 3, 4. e No inversion occurring after January 1, 2006 (when data on O_3 were available), lasted longer than 8 days. f OR for ordinal variable coded using median category scores: 0, 2, 5, 8, 15. g OR for ordinal variable coded using median category scores: 0, 2, 5, 8, 18.

References

Alduchov OA, Eskridge RE. 1996. Improved Magnus' form approximation of saturation vapor pressure. J Appl Meteor 35:601–609.

Lawrence MG. 2005. The relationship between relative humidity and the dewpoint temperature in moist air: a simple conversion and applications. Bull Am Meteor Soc 86:225-233.
